# Multiplexed methylation profiles of tumor suppressor genes and clinical outcome in lung cancer

**DOI:** 10.1186/1479-5876-8-86

**Published:** 2010-09-17

**Authors:** Mónica Castro, Laura Grau, Patricia Puerta, Liliana Gimenez, Julio Venditti, Silvia Quadrelli, Marta Sánchez-Carbayo

**Affiliations:** 1 Tumor Markers Group, Molecular Pathology Program, Spanish National Cancer Center, Madrid, Spain; 2 Oncology Department, Instituto Angel H. Roffo, Buenos Aires, Argentina; 3 Oncology Department, Hospital Británico, Buenos Aires, Argentina

## Abstract

**Background:**

Changes in DNA methylation of crucial cancer genes including tumor suppressors can occur early in carcinogenesis, being potentially important early indicators of cancer. The objective of this study was to examine a multiplexed approach to assess the methylation of tumor suppressor genes as tumor stratification and clinical outcome prognostic biomarkers for lung cancer.

**Methods:**

A multicandidate probe panel interrogated DNA for aberrant methylation status in 18 tumor suppressor genes in lung cancer using a methylation-specific multiplex ligation-dependent probe amplification assay (MS-MLPA). Lung cancer cell lines (n = 7), and primary lung tumors (n = 54) were examined using MS-MLPA.

**Results:**

Genes frequently methylated in lung cancer cell lines including SCGB3A1, ID4, CCND2 were found among the most commonly methylated in the lung tumors analyzed. HLTF, BNIP3, H2AFX, CACNA1G, TGIF, ID4 and CACNA1A were identified as novel tumor suppressor candidates methylated in lung tumors. The most frequently methylated genes in lung tumors were SCGB3A1 *and DLC1 *(both 50.0%). Methylation rates for ID4, DCL1, BNIP3, H2AFX, CACNA1G and TIMP3 were significantly different between squamous and adenocarcinomas. Methylation of RUNX3, SCGB3A1, SFRP4, and DLC1 was significantly associated with the extent of the disease when comparing localized versus metastatic tumors. Moreover, methylation of HTLF, SFRP5 and TIMP3 were significantly associated with overall survival.

**Conclusions:**

MS-MLPA can be used for classification of certain types of lung tumors and clinical outcome prediction. This latter is clinically relevant by offering an adjunct strategy for the clinical management of lung cancer patients.

## Background

Lung cancer is the third most frequent tumor, representing the leading cause of cancer death [1]. Non-small cell lung cancer (NSCLC) is the most common variant. NSCLC is the superseding term for various types of lung cancer such as the most common ones, adenocarcinomas and squamous carcinomas [2-4]. Even within patients at the earliest stages of the disease, a significant number recur after therapeutic surgery and adjuvant chemotherapy, and ultimately die from their disease. Lung cancer cure rate remains disappointing, with five-year survival rates limited to 15-20% [1]. Understanding the molecular basis of lung cancer will enable the identification of high-risk populations for effective early detection, and prognostic and predictive markers of tumor behaviour.

Lung cancer can be described as a molecular disease, driven by the multistep accumulation of genetic, epigenetic and environmental factors, among others [5,6]. Epigenetic alterations, including DNA methylation, histone modifications, and miRNAs may result in silencing of cancer-related genes. Alterations of DNA methylation patterns have been recognized as the most common epigenetic events in human cancers. Aberrant methylation of normally unmethylated CpG-rich areas, also known as CpG islands, located in or near the promoter region of many genes, has been associated with the initiation and progression of several types of cancer [7-11]. In NSCLC, transcriptional inactivation of important tumor suppressor, DNA repair, and metastasis inhibitor genes, among others, has been reported [2,12]. Therefore, the detection of aberrant promoter methylation of cancer-related genes may be essential for the diagnosis, prognosis and/or detection of metastatic potential of tumors, including lung cancer. As the number of genes methylated in cancer is large and increasing, sensitive and robust multiplexed methods for detecting of aberrant methylation of promoter regions are therefore, desirable.

Historically, the molecular pathogenesis of cancer has been analyzed one gene at a time. CpG arrays represent a high-throughput technology accelerating the discovery of genes frequently hypermethylated during disease progression, also for lung cancer [13,14]. Methylation specific multiplex ligation-dependent probe amplification (MS-MLPA) is a PCR-based technique allowing the semiquantitative detection of changes in DNA promoter methylation of multiple genes in a single experiment [15,16]. Discrimination between methylated and unmethylated targets is based on the annealing of probes containing a recognition site for the methylation-sensitive restriction enzyme HhaI. MS-MLPA has been applied to the multiplexed measurement of methylated genes in several diseases, including cancer [17-28]. Its potential utility in lung cancer has not been characterized. In this study, we initially assessed, by MS-MLPA, whether a selected panel of candidate tumour suppressor genes could be methylated in lung cell lines and tumors. Next, we determined whether the methylation status of such genes could contribute for lung tumor stratification and clinical outcome prognosis.

## Methods

### Lung cancer cell lines

Six NSCLC cell lines consisting of three adenocarcinoma cell lines (A549, H522 and H358), two large carcinoma cell lines (H460, H661), and one squamous cell carcinoma cell line (H226), as well as one small cell lung cancer cell line (SCLC) (H841) were obtained from the American Type Culture Collection (Rockville, MD, US), grown in RPMI-1640 medium (Sigma) supplemented with 10% fetal bovine serum, and collected under standard tissue culture protocols. The lung cancer cell line, H460, was included in all sample runs in order to test the reproducibility of the MS-MLPA test.

### Tumor samples

The study cohort consisted of a series of archived paraffin-embedded blocks from 54 NSCLC patients. Patients with local disease (stage I to resectable stage III) were treated surgically and those with advanced disease (stage III and IV) received systemic and/or local treatment. Primary lung tumors were collected after institutional review board approval and handled anonymously following ethical and legal protection guidelines of human subjects. The observation period ranged from 2 to 79 months, with a median follow-up of 20,5 months. Inclusion criteria of newly diagnosed lung cancer patients were based on the histopathologic information, covering from early to advanced stages. It was also required to have tissue material available for obtaining high-quality DNA for methylation analyses. Of the 54 NSCLC patients, 22 had TNM Stage I-II, 18 had Stage III, and 14 stage IV defined under standard criteria [29]. The tumors were histologically classified as adenocarcinomas (n = 32), squamous cell carcinomas (n = 21) and large cell carcinoma (n = 1) according to the histological typing of lung tumors of the World Health Organization [29]. Demographic and clinicopathologic information of the lung cases analyzed is described in Table 1.

**Table 1:** Demographic and clinicopathologic information of the lung cases analyzed.

	Clinical Parameters	Cases n (%)
**Age**	≥65	28 (51.9%)
	<65	26 (48.1%)

**Gender**	Male	37 (68.5%)
	Female	17 (31.5%)

**Smoking history**	Yes	46 (85%)
	No	6 (11%)
	Unknown	2 (3%)

**Karnosfsky**	≥80	45 (83.3%)
	<80	9 (16.7%)

**Histology**	Squamous Cell Carcinoma	21 (38.5%)
	Adenocarcinoma	32 (59.6%)
	Large Cell Carcinoma	1 (1.9%)

**Differentation grade**	Good differentiated	11 (20.4%)
	Moderate	16 (29.6%)
	Poor	27 (50%)

**Stage**	I-II	22 (40.7%)
	III	18 (33.3%)
	IV	14 (26%)

**Local/Advanced**	Local (stage I II)	22 (40.7%)
	Advanced (stage III-IV)	32 (59.3%)

**Progression**	Yes	26 (48.1%)
	No	28 (51.9%)

**Death**	Yes	25 (46.3%)
	No	29 (53.7%)

Summary of the distribution of the following variables among patients studied: age, gender, smoking history, Karnofsky status, histology, differentiation grade, stage, extension of disease, and the number of cases progressing or dying of the disease during the study.

### DNA extraction

Genomic DNA from cell lines and tissue was extracted using standard methods. Paraffin-embedded tissues were macro-dissected based on hematoxylin-eosin evaluations to ensure a minimum of 75% of tumor cells [30]. Corresponding slides were digested using proteinase K (Roche Diagnostics GmbH, Mannheim, Germany) overnight before DNA extraction. Concentration and purity of DNA samples were determined with a ND-1000 spectrophotometer (NanoDrop Technologies, Wilmington, DE, USA). DNA quality was evaluated based on 260/280 ratios of absorbances and the integrity was also checked by gel electrophoresis analysis using the Agilent 2100 Bioanalyzer (Agilent Technologies, Palo Alto CA).

### Methylation-Specific Multiplex Ligation-Dependent Probe Amplification (MS-MLPA)

The present study used the MS-MLPA probe set ME003 (MRC-Holland, Amsterdam, The Netherlands) which can simultaneously check for aberrant methylation at one or two CpG dinucleotides of the following proven or suspected 18 tumor suppressor genes (Table 2). Probe sequences, gene loci and chromosome locations can be found at http://www.mlpa.com (date of accession: 25-May-2010). Several genes were evaluated by two probes, which recognized different Hha1 restriction sites in their promoter regions. The experimental procedure was carried out and results analyzed according to the manufacturer's instructions, with minor modifications. In short, DNA (200 ng) was dissolved up to 5 μl TE-buffer (10 mM Tris pH 8.2, 1 mM EDTA pH 8.0), denatured and subsequently cooled down to 25°C. After adding the probe mix, the probes were allowed to hybridize (16 h at 60°C). Subsequently, the samples were divided in two: one half of the samples were ligated, whereas for the other part of the samples, ligation was combined with the HhaI digestion enzyme. This digestion resulted in ligation of only the methylated sequences. PCR was performed on both parts of the samples in a volume of 50 μl containing 10 μl of the ligation reaction mixture using a thermal cycler (MJ Research Inc., Waltham, MA, USA), with 35 cycles of denaturation at 95°C for 30 s, annealing at 60°C for 30 s and extension at 72°C for 1 min with a final extension of 20 min at 72°C. Aliquots of 2 μl of the PCR reaction were combined with 0.12 μl LIZ-labeled internal size standard (Applied Biosystems, Foster City, CA, USA) and 9.0 μl deionized formamide. After denaturation, fragments were separated and quantified by electrophoresis on an ABI 3700 capillary sequencer and the Peak Scanner v1.0 analysis software (both Applied Biosystems). Peak identification and values corresponding to peak size in base pairs (bp), and peak areas were used for further data processing. Automated fragment and data analysis was performed exporting the peak areas to an excel-based analysis program (Coffalyser V8, MRC-Holland). For hypermethylation analysis the 'relative peak value' or the so-called 'probe fraction' of the ligation-digestion sample is divided by the 'relative peak value' of the corresponding ligation (undigested) sample, resulting in a so-called 'methylation-ratio' (M-ratio). Aberrant methylation was scored when the calculated M-ratio was ≥0.30, corresponding to 30% of methylated DNA. The methylated ratios were interpreted as absence of hypermethylation (0.00-0.29), mild hypermethylation (0.30-0.49), moderate hypermethylation (0.50-0.69), and extensive hypermethylation (>0.70). In genes with more than one probe, their ratios were calculated independently for methylation analysis.

**Table 2:** Information of the tumor suppressor genes analyzed.

Gene	**Name**^**a**^	Probes	Functional implications	**Chromosomal Localization**^**b**^
*PRDM2*	PR domain containing 2, with ZNF domain	09146-L02862	Cell cycle control	1p36

*RUNX3*	Runt-related transcription factor 3	11131-L03905	TGFB signaling	1p36

*RARB*	Retinoic acid receptor beta	10362-L10900	Cell differentiation and proliferation	3p24

***HLTF***	Helicase-like transcription factor	09152-L09384(probe 1) 02758-L02207(probe 2)	Transcription regulation	3q25.1-q26.1

*SCGB3A1*	Secretoglobin, family 3A, member 1	03305-L09382 (probe1) 11132-L12956(probe 2)	Cell differentiation and proliferation	5q35-qter

***ID4***	Inhibitor of DNA binding 4, dominant negative helix-loop-helix protein	04497-L03909(probe 1) 04496-L03908(probe 2)	Transcription regulation	6p22.3

*TWIST1*	Twist homolog 1 (Drosophila)	02080-L02886	Cell differentiation and proliferation	7p21

*SFRP4*	Secreted frizzled-related protein 4	03744-L03204(probe 1) 09147-L03205(probe 2)	WNT antagonism	7p14.1

*DLC1*	Deleted in liver cancer 1	02754-L02203(probe 1) 02753-L02202(probe 2)	Cell differentiation and proliferation	8p22

*SFRP5*	Secreted frizzled-related protein 5	09149-L03207(probe 1) 09148-L12957(probe 2)	WNT antagonism	10q24

***BNIP3***	BCL2/adenovirus E1B 19kDa Interacting protein 3	07138-L12958	Proliferation and apoptosis	10q.26.3

***H2AFX***	H2A histone family, member X	08511-L08607(probe 1) 08509-L08605(probe 2)	Transcription regulation	11q23.3

*CCND2*	Cyclin D2	03313-L02668(probe 1) 03312-L09381(probe 2)	Cell cycle control	12p13

***CACNA1G***	Calcium channel, voltage-dependent, T type, alpha 1G subunit	10123-L10466	Cell differentiation and proliferation	17q22

***TGIF***	TGFB-induced factor homebox 1	02850-L13256	TGFB signaling	18p11.31

*BCL2*	B-cell CLL/lymphoma2	10352-L10890	Proliferation and apoptosis	18q21.3

***CACNA1A***	Calcium channel, voltage-dependent, P/Q type, alpha 1A subunit	09055-L09224	Cell differentiation and proliferation	19p13

*TIMP3*	TIMP metallopeptidase inhibitor 3	10357-L10895(probe 1) 10354-L10892(probe 2)	Invasion and metastasis	22q12.3

Summary of the gene description, probes, functional implications, and chromosomal location of the tumor suppressor genes analyzed in this study.

Gene names in bold highlight novel candidates never reported to be methylated in lung cancer to date. a. Human Genome Organization Nomenclature; b. Approved gene name from Human Genome Organization available at http://www.genenames.org/

### Statistical Analysis

Coefficients of variation for each probe were estimated based on the ratio of the standard deviation and the respective mean of four replicates of the H460 cell line. Associations among MS-MLPA methylation and tumor stage and grade were evaluated using non-parametric Wilcoxon-Mann-Whitney and Kruskall-Wallis tests using Bonferroni adjustment for multiple testing. Associations between methylation candidates were analyzed using Kendall's tau ß test, considering only two-sided p-values 0.05 to be statistically significant. For each probe of the assay, methylation was scored when the calculated M-ratio was ≥0.30. Associations of methylation of each gene probe with overall survival were also evaluated using the log-rank test in those cases for which follow-up information were available. Overall survival time was defined as the months elapsed between surgery and death as a result of disease (or the last follow-up date). Patients who were alive at the last follow-up or lost to follow-up were censored. Survival curves were plotted using the standard Kaplan-Meier methodology [31]. Statistical analyses were performed using the SPSS statistical package (SPSS 17.0.1 for Windows 2009, Chicago, IL, USA).

## Results

### Quality assessment of MS-MLPA assay

In order to test the reproducibility of the assay, a lung cancer cell line, H460, was included as control samples each assay run. The methylation ratios of these replicated experiments of the cell line analyzed and their coefficients of variation are shown in additional file 1, Table S1. Using the thresholds defined above for methylation detection suggested high reproducibility of the methylation profiles. In conclusion, these initial analyses revealed reproducible results allowing methylation assessment using the selected panel of candidate genes.

### MS-MLPA profiles of lung cancer cell lines

The methylation profiles of the 18 genes under study were initially tested in seven cell lines derived from lung tumors of different histopathologic variants. Additional file 2, Table S2 provides an overview of the methylation patterns of these cell lines grouped based on the histopathology of the tumors from which these lung cancer cell lines were derived from. The percentual methylation for each gene is provided as well. Several genes: SCGB3A1, ID4, SFRP5, CCND2, and CACNA1A, were found methylated in at least 4 out of the 7 cell lines analyzed, covering various histopathologic types. These initial analyses suggested that the panel of candidate genes selected could be appropriate to detect aberrant methylation profiles in human lung tumors.

### MS-MLPA profiles for clinico-histopathologic stratification of lung tumors

**Figure 1:**
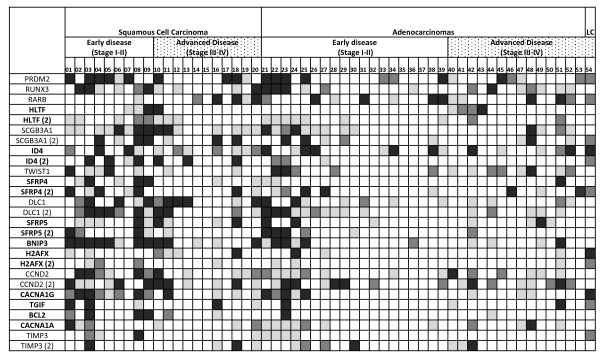
**Methylation profiles of lung tumors**. The methylated ratios were interpreted as absence of hypermethylation (0.00-0.29), highlighted as white cells; mild hypermethylation (0.30-0.49) highlighted as light grey cells; moderate hypermethylation (0.50-0.69), highlighted as medium grey cells; and extensive hypermethylation (0.70-1.00), highlighted as dark grey cells. Gene names in bold highlight novel candidates never reported to be methylated in lung cancer to date. Advanced tumors are highlighted with dots. LC: large cell carcinomas.

In the next step, we tested whether MS-MLPA could be applied to lung tumors (Figure 1, Table 3). Overall, the most frequent hypermethylated genes found by MS-MLPA were DLC1 (50%), SCGB3A1 (50.0%), CCND2 (48.1%), ID4 (46.3%), BNIP3 (44.4%), RUNX3 (42.5%), and PRDM2 (40.7%). Notably, genes methylated in lung tumor specimens frequently overlapped with those found to be methylated in the lung cancer cell lines as shown above. Promoter hypermethylation of genes previously reported methylated in lung cancer included PRDM2, RUNX3, RARB, SCGB3A1, TWIST1, SFRP4, DLC1, SFRP5, CCND2, BCL2 and TIMP3 (Reviewed in additional file 3, Table S3). Methylation was newly identified for HLTF, ID4, BNIP3, H2AFX, CACNA1G, CACNA1A, and TGIF. The percentual methylation rates of each gene depending on the different clinicopathologic variables are shown in Table 3. The genes more frequently methylated in adenocarcinomas were: RARB, TWIST1, and CACNA1A, while the most commonly methylated genes in squamous tumors were SCGB3A1, ID4, SFRP4, SFRP5, DCL1, BNIP3, H2AFX, CACNA1G, TGIF, TIMP3 and BCL2. Statistically significantly different methylation rates were observed for ID4-2 (p = 0.011), DCL1 (p = 0.019), BNIP3 (p = 0.003), H2AFX (p = 0.001), H2AFX-2 (p = 0.005), CACNA1G (p = 0.007) and TIMP3 (p = 0.021) when comparing squamous versus adenocarcinoma cases. When comparing methylation rates in localized tumors versus metastatic disease, the methylation of RUNX3 (p = 0.013), SCGB3A1-2 (p = 0.008), SFRP4-2 (p = 0.022), and DLC1 (p = 0.016) was significantly associated with the presence of metastatic disease. The methylation of the same genes was also associated with tumor stage RUNX3 (p = 0.040), SCGB3A1 (p = 0.032), SFRP4 (p = 0.033), and DLC1 (p = 0.035). RARB methylation (p = 0.028) was associated with the Karnofsky status. Methylation of several genes was simultaneously present in the lung tumors analyzed, as revealed by Kendall's tau correlations shown in additional file 4, Table S4. We did not find any significant association between methylation of the genes under study and age, gender, smoking history (data not shown). Methylation rates regarding histology lung subtypes, differentiation grade and tumor stage (comparing localized versus advanced disease), are provided in Table 3. In conclusion, this set of analyses suggested that the panel of candidate genes selected could be of clinical relevance for the clinicopathologic staging of human lung tumors.

**Table 3:** Summary of the frequency of methylation of the genes in lung tumors based of their main clinicopathologic variables.

Gene	Overall (%)	Histology (%)		Differentation Grade (%)			Tumor stage (%)	
	Methylation n = 54	SCC n = 21	ADC n = 32	Good n = 11	Moderate n = 16	Poor n = 27	Local n = 22	Advanced n = 32
*PRDM2*	22 (40.7)	10 (45.4)	12 (37.5)	21 (45.6)	0 (0)	12 (44.4)	10 (45.4)	12 (37.5)

*RUNX3*	23 (42.5)	13 (59.1)	10 (31.2)	19 (41.3)	2 (33.3)	10 (37.0)	13 (59.1)	10 (31.2)

*RARB*	20 (37)	8 (36.4)	12 (37.5)	17 (36.9)	3 (50.0)	10 (37.0)	8 (36.4)	12 (37.5)

***HLTF***	8 (14.8)	1 (4.5)	7 (21.9)	8 (17.4)	0 (0)	7 (25.9)	1 (4.5)	7 (21.9)

***HLT-2***	17 (31.5)	9 (40.9)	8 (25.0)	14 (30.4)	2 (33.3)	10 (37.0)	9 (40.9)	8 (25.0)

*SCGB3A1*	27 (50.0)	15 (68.2)	12 (37.5)	24 (52.2)	2 (33.3)	15 (55.5)	15 (68.2)	12 (37.5)

*SCGB3A1-2*	17 (31.5)	12 (54.5)	5 (15.6)	12 (26.0)	4 (66.7)	9 (33.3)	12 (54.5)	5 (15.6)

***ID4***	25 (46.3)	12 (54.5)	13 (40.6)	23 (50.0)	1 (1.7)	12 (44.4)	12 (54.5)	13 (40.6)

***ID4-2***	11 (20.4)	5 (22.7)	6 (18.7)	10 (21.7)	1 (1.7)	7 (25.9)	5 (22.7)	6 (18.7)

*TWIST1*	21 (38.9)	9 (40.9)	12 (37.5)	19 (41.3)	2 (33.3)	10 (37.0)	9 (40.9)	12 (37.5)

*SFRP4*	10 (18.5)	7 (31.8)	3 (9.4)	9 (19.5)	1 (1.7)	5 (18.5)	7 (31.8)	3 (9.4)

*SFRP4-2*	16 (29.6)	11 (50.0)	5 (15.6)	12 (26.0)	3 (50.0)	8 (29.6)	11 (50.0)	5 (15.6)

*DLC1*	22 (40.7)	12 (54.5)	10 (31.2)	18 (39.1)	3 (50.0)	13 (48.1)	12 (54.5)	10 (31.2)

*DLC1-2*	27 (50.0)	15 (68.2)	12 (37.5)	21 (45.6)	5 (83.3)	14 (51.8)	15 (68.2)	12 (37.5)

*SFRP5*	17 (31.5)	8 (36.4)	9 (28.1)	15 (32.6)	2 (33.3)	10 (37.0)	8 (36.4)	9 (28.1)

*SFRP5-2*	12 (22.2)	6 (27.3)	6 (18.7)	9 (19.6)	2 (33.3)	8 (29.6)	6 (27.3)	6 (18.7)

***BNIP3***	24 (44.4)	12 (54.5)	12 (37.5)	20 (43.5)	2 (33.3)	13 (48.1)	12 (54.5)	12 (37.5)

***H2AFX***	10 (18.5)	5 (22.7)	5 (15.6)	9 (19.5)	1 (1.7)	6 (22.2)	5 (22.7)	5 (15.6)

***H2AFX-2***	9 (16.7)	6 (27.3)	3 (9.4)	8 (17.4)	1 (1.7)	5 (18.5)	6 (27.3)	3 (9.4)

*CCND2*	26 (48.1)	13 (59.1)	13 (40.6)	22 (47.8)	3 (50.0)	15 (55.5)	13 (59.1)	13 (40.6)

*CCND2-2*	29 (53.7)	14 (63.6)	15 (46.9)	25 (54.3)	4 (66.7)	15 (55.5)	14 (63.6)	15 (46.9)

***CACNA1G***	21 (38.9)	12 (54.5)	9 (28.1)	19 (41.3)	1 (1.7)	12 (44.4)	12 (54.5)	9 (28.1)

***TGIF***	10 (18.5)	5 (22.7)	5 (15.6)	9 (19.5)	1 (1.7)	6 (22.2)	5 (22.7)	5 (15.6)

*BCL2*	8 (14.8)	4 (18.2)	4 (12.5)	8 (17.4)	0 (0)	5 (18.5)	4 (18.2)	4 (12.5)

***CACNA1A***	18 (33.3)	11 (50.0)	7 (21.9)	13 (28.3)	4 (66.7)	8 (29.6)	11 (50.0)	7 (21.9)

*TIMP3*	10 (18.5)	7 (31.8)	3 (9.4)	9 (19.6)	1 (1.7)	5 (18.5)	7 (31.8)	3 (9.4)

*TIMP3-2*	11 (20.4)	7 (31.8)	4 (12.5)	9 (19.6)	1 (1.7)	6 (22.2)	7 (31.8)	4 (12.5)

The number of samples (n) displaying a methylation ratio higher than 0.3, as well as their percentual frequency within each group of specimens under analyses was included.

Highlighted genes in bold represented novel candidates never reported methylated in lung cancer to date. Ys: years; SCC: squamous cell carcinoma; ADC: adenocarcinoma.

### MS-MLPA profiles for clinical outcome prognosis for lung cancer patients

**Figure 2:**
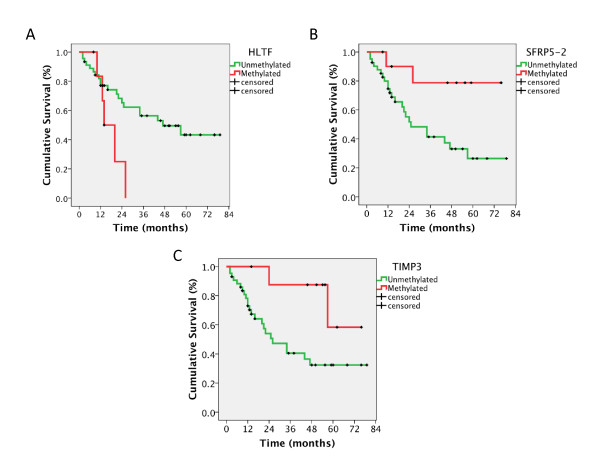
**Methylation profiles as clinical outcome prognosticators for lung cancer patients**. **A) **Kaplan-Mayer curve survival analysis indicating that tumors methylated for HTLF showed poor survival than those unmethylated for this gene (log rank, p = 0.035). **B) **Kaplan-Mayer curve survival analysis indicating that tumors methylated for SFRP5-2 (probe 2) showed better survival than those unmethylated for this gene (log rank, p = 0.021). **C) **Kaplan-Mayer curve survival analysis indicating that tumors methylated for TIMP3 showed better survival than those unmethylated for this gene (log rank, p = 0.030).

In the next step, we tested whether MS-MLPA could be applied to differentiate patients with different clinical outcome, using overall survival as the clinical endpoint. We observed that patients with tumors methylated for the HTLF gene showed an overall survival significantly shorter as compared to patients unmethylated for HTLF (log rank, p = 0.035; Figure 2A). In contrast, survival was significantly longer in patients with methylation for SFRP5 (probe 2) (log rank, p = 0.021; Figure 2B); and TIMP3 (log rank, p = 0.030; Figure 2C), as compared to patients with no aberrant methylation of these genes. Importantly, this set of analyses indicated that the methylation of three genes was significantly associated with overall survival, suggesting that the panel of candidate genes under analyses could be of clinical relevance as prognosticators of the clinical outcome of patients affected with lung tumors.

## Discussion

The present study evaluates the application of a multiplexed methylation technique in lung cancer. MS-MLPA was initially tested in cell lines and tissue specimens representing different steps of lung cancer progression supporting the panel of the tumor suppressor genes selected to be altered in lung cancer. In this study, we included genes with important roles in cell cycle control (PRDM2, CCND2), transcription regulation (HTLF, ID4, H2AFX), TGF-β signaling (RUNX3, TGIF), WNT antagonism (SFRP4, SFRP5), cell differentiation and proliferation (SCGB3A1, TWIST1, RARB, CACNA1A, CACNA1G, DLC1), proliferation and apoptosis (BNIP3, BCL2), and invasion and metastasis (TIMP3). Our present data is in concordance with previous reports showing altered methylation patterns in lung cancer in genes such as PRDM2 [32], RUNX3 [33-38], RARB [37,39-41], SCGB3A1 [42,43], TWIST1 [44], DLC1 [45,46], SFRP4 [36,44], SFRP5 [36,38,44,47], CCND2 [40,41,48,49], BCL2 [50] and TIMP3 [14,46,51]. Importantly, our study identified seven novel methylated candidates in lung cancer, including HLTF, ID4, BNIP3, H2AFX, CACNA1G, CACNA1A and TGIF. The clinical outcome of the patients whose tumors were analyzed using this technique revealed that individual tumors behaved according to histopathologic staging and also to their methylation patterns analyzed using this type of multiplexed strategy. The MS-MLPA approach thereby offered an opportunity to test and improve histopathologic stratification and also prognostic statements. This latter is clinically relevant since it offers an alternative adjunct strategy for the clinical management of patients affected with lung cancer.

Among the high-throughput techniques available today for epigenetic alterations assessment, the CpG array represents the main comprehensive platform already applied to identify methylation candidates in lung cancer [13,14]. To our knowledge, the multiplexed MS-MLPA technique has not been employed to analyze the methylation profiles in lung cancer. The advantages of MS-MLPA technique as an alternative for MS-PCR include: allowing screening of multiple predefined promoter methylation candidates in one experiment using a low amount of DNA (100-200 ng), being feasible using DNA extracted from tissue (even in formalin fixed material), providing semiquantitative data, and requiring only standard laboratory equipment. Furthermore, the (potentially) troublesome bisulfite conversion of unmethylated cytosines required for MS-PCR can be omitted in MS-MLPA using a methylation-sensitive digestion. Methylation indices for the majority of the probes under study were consistent and reproducible. Overall, the variation of the methylation ratios obtained for each probe revealed inter-assay reproducibility reliable enough for clinical practice.

The identification of the different methylation profiles in lung cancer cell lines provided first insights of the potential impact of these candidate genes for human lung cancer. Results of the tumor set for the top differentiating genes concurred with the main MS-MLPA results in the cells set (supporting the cancer specificity of the methylated candidates), and also with previous reports describing methylation for some of the candidates under study, such as PRDM2 [32], RUNX3 [35,36], SFRP4 and SFRP5 [36], SCGB3A1 [43], DLC1 [46], CCND2 [48]. In our series, RUNX3 [33-35,38], SCGB3A1 [43], CCND2 [49] exhibited higher methylation rates as compared to these reports; whereas SFRP4 and SFRP5 [44], DLC1 [45], TIMP3 [51] showed lower methylation rates than previous studies. In addition to the inter-individual variation, these differences could be attributed to several issues: 1) it is important to be aware that aberrant methylation needs to meet the cutoff ratio of 30% or greater set by the mathematical algorithm designed to distinguish legitimate methylation peaks. Variation in cutoff setting would render improved accuracies for each specific gene. 2) Discrepancy in the frequency of methylation might be attributed in part to the number and type of stages analyzed. 3) Heterogeneity of the promoter methylation may exist within the individual gene promoters for certain genes in lung cancer carcinomas. MS-MLPA is only based on a single CpG site compared to an average of 4-6 CpG sites in MS-PCR assays. Since only a small part of the promotor is usually analyzed by MS-MLPA, the methylation of additional of nearby CpG islands cannot be excluded. 4) Availability of two probes targeting different CpG islands with different methylation ratios for two of the genes analyzed served to highlight the differential methylation and potential consequences of each specific CpG site within a gene. The relative impact of each site was observed for those genes for which different probes were included targeting different CpG sites displaying different methylation rates. 5) MS-MLPA ratios may potentially be underestimated due to the presence of normal (U) 'contaminating' cells in the tumor sample. However, whereas the detection of an unmethylated promoter next to methylated sequences is usually disregarded as originating from normal tissue, it may frequently reflect tumor heterogeneity and the polyclonality of the tumors regarding hypermethylation.

Despite of not containing a lung cancer specific panel of tumor suppressors, we observed correlation of hypermethylation with lung cancer histopathologic variables. We detected distinct methylation profiles between squamous and adenocarcinomas, in concordance with previous reports evaluating part of the genes analyzed using MS-PCR methods [33-37,41-45,48,51]. Among the novel methylated candidates identified, ID4, BNIP3, H2AFX, CACNA 1G, TGIF were more frequently methylated in squamous tumors, while HTLF and CACNA1A were commonly methylated in adenocarcinomas. In agreement with previous observations, methylation of RUNX3 [36,38], SCGB3A1 [42], DLC1 [45] and SFRP4 [36,44], was identified as early events associated with early differentiation and stage. The presence of different methylation patterns in different tumor stages supports the notion that epigenetic events may be involved in tumor progression, after the accumulation of additional genomic instability, and other epigenetic and genetic events [5,36]. Kendall's tau associations revealed the frequent simultaneous methylation of the genes analyzed, especially for TIMP3 and H2AFX, SFRP5 and HTLF, and CACNA1G and BNIP3. These observations highlight how epigenetic regulation impact on different cancer genes carrying out critical cell functions in neoplastic cells. Importantly, the methylation of three of the genes analyzed (HTLF, SFRP5 and TIMP3) was associated with clinical outcome. Hypermethylation of HTLF was associated with poor survival, in agreement with previous studies indicating the silencing of the gene by methylation predicting colorectal cancer recurrence [52]. On the other hand, hypermethylation of SFRP5 and TIMP3 was associated with improved survival. TIMP3 methylation was also previously found associated with better survival in NSCLC [51], and bladder cancer [53]. These findings are clinically relevant for the adjunct potential of the methylation assessment of any of these three to identify lung cancer patients more likely to show a poor clinical behavior. Since the biology and the mechanisms by which these genes play a tumor suppressor role is not fully characterized, and due to the limited number of cases analyzed, the interpretation of the prognostic significance of their promoter hypermethylation may warrant further investigation.

## Conclusions

MS-MLPA allowed identification of a number of new and possibly interesting epigenetic alterations such as HLTF, ID4, BNIP3, H2AFX, CACNA1G, CACNA1A and TGIF genes, serving to gain more insight into the development of lung carcinomas. This report highlights that the identification of aberrant methylation in promoter regions of cancer genes yields important tumor biomarkers, underscoring a role for epigenetics in the early pathogenesis of the major histological subtypes of lung cancer. The innovative applicability of MS-MLPA in the types of samples analyzed contributed to the further understanding of lung cancer biology. To what extent these genes contribute or are functionally involved in the different steps during tumorigenesis and cancer progression remains to be determined. These genes would represent attractive targets for cancer therapy, given the reversible nature of epigenetic gene silencing. Importantly, the clinical translational applications of the MS-MLPA platform using tissue paraffin/embedded material relate not only to adjunct tumor classification, but also for clinical outcome prognosis. The general character of the assay used (with predefined tumor suppressor genes not necessary specific to any tumor type) suggested the need to investigate regions that would be more relevant for lung cancer and to develop targeted tumor-specific customized MS-MLPA assays. Considering that the mortality of lung cancer could be greatly reduced through detection of the disease at the earliest stages, in the near future, the semiquantitative aspect of MS-MLPA may prove to play a role not only for clinical outcome prognosis and risk stratification but may also aid for early detection and follow-up of lung cancer patients, and predict therapeutic response.

## Abbreviations

ADC: adenocarcinoma; *BCL2: *B-cell CLL/lymphoma 2; *BNIP3: *BCL2/adenovirus E1B 19 kDa Interacting protein 3; *CACNA1A: *Calcium channel, voltage-dependent, P/Q type, alpha 1A subunit; *CACNA1G: *Calcium channel, voltage-dependent, T type, alpha 1G subunit; *CCND2: *Cyclin D2; *DLC1: *Deleted in liver cancer 1; *H2AFX: *H2A histone family, member X; *HLTF*: Helicase-like transcription factor; *ID4: *Inhibitor of DNA binding 4, dominant negative helix-loop-helix protein; LC: Large cell carcinoma; MS-MLPA: Methylation-Specific Multiplex Ligation-Dependent Probe Amplification Assay; NSCLC: Non-Small Cell Lung Cancer; *PRDM2: *PR domain containing 2, with ZNF domain; *RARB: *Retinoic acid receptor beta; *RUNX3: *Runt-related transcription factor 3; SCC: squamous cell carcinoma; *SCGB3A1*: Secretoglobin, family 3A, member 1; SCLC: Small Cell Lung Cancer; *SFRP4: *Secreted frizzled-related protein 4; *SFRP5: *Secreted frizzled-related protein 5; *TGIF: *TGFB-induced factor homebox 1; *TIMP3: *TIMP metallopeptidase inhibitor 3; *TWIST1: *Twist homolog 1 (Drosophila); Ys: Years.

## Competing interests

The authors declare that they have no competing interests.

## Authors' contributions

MC participated in acquiring clinical and laboratory data, data analysis and interpretation, and drafted the manuscript. PP and LG participated in acquiring clinical and laboratory data, data analysis and data interpretation and drafted the manuscript. LG, JV, and SQ participated in acquiring clinical samples and follow-up clinical information. MSC participated in study design and coordination, data analysis and interpretation and final writing of the manuscript. All authors read and approved the final manuscript.

## Supplementary Material

Additional file 1 **Table S1**: **Quality assessment of methylation profiles**: Inter-assay reproducibility including coefficient of variations among replicates of each probe for the lung control cell line. The methylated ratios were interpreted as absence of hypermethylation (0.00-0.29), highlighted as white cells; mild hypermethylation (0.30-0.49) highlighted as light grey cells; moderate hypermethylation (0.50-0.69), highlighted as medium grey cells; and extensive hypermethylation (0.70-1.00), highlighted as dark grey cells. Gene names in bold highlight novel candidates never reported to be methylated in lung cancer to date. Click here for file

Additional file 2 **Table S2**: **Methylation profiles of lung cancer cell lines**. The methylated ratios were interpreted as absence of hypermethylation (0.00-0.29), highlighted as white cells; mild hypermethylation (0.30-0.49) highlighted as light grey cells; moderate hypermethylation (0.50-0.69), highlighted as medium grey cells; and extensive hypermethylation (0.70-1.00), highlighted as dark grey cells. Gene names in bold highlight novel candidates never reported to be methylated in lung cancer to date. Cell lines derived from metastatic tumors are highlighted with dots. SCC: squamous cell carcinoma; LC: large cell carcinoma; SCLC: small cell lung cancer Click here for file

Additional file 3 **Table S3**: **Complementary information of the genes analyzed using MS-MLPA**. Review of the functional implications and methylation studies of the candidate genes analyzed in this study in lung cancer. Click here for file

Additional file 4 **Table S4: Kendall's tau correlation coefficients evaluating associations among the candidate genes**. Two sided significant coefficients are highlighted in grey. Click here for file
