# Supplementary material for: Multiplexed methylation profiles of tumor suppressor genes and clinical outcome in lung cancer
Source: J Transl Med. 2010 Sep 17;8:86. doi: 10.1186/1479-5876-8-86 (PMC2955578; doi:10.1186/1479-5876-8-86)
Supplement: Additional file 2 — Table S2: Methylation profiles of lung cancer cell lines. The methylated ratios were interpreted as absence of hypermethylation (0.00-0.29), highlighted as white cells; mild hypermethylation (0.30-0.49) highlighted as light grey cells; moderate hypermethylation (0.50-0.69), highlighted as medium grey cells; and extensive hypermethylation (0.70-1.00), highlighted as dark grey cells. Gene names in bold highlight novel candidates never reported to be methylated in lung cancer to date. Cell lines derived from metastatic tumors are highlighted with dots. SCC: squamous cell carcinoma; LC: large cell carcinoma; SCLC: small cell lung cancer [file 1479-5876-8-86-S2.DOC]

| **Gene** | **Methylation**  **n (%)** | **SSC** | **Adenocarcinomas** | | | **LCC** | | **SCLC** |
| --- | --- | --- | --- | --- | --- | --- | --- | --- |
|  |  | **H226** | **H368** | **A549** | **H522** | **H460** | **H661** | **H841** |
| **PRDM2** | 1 (14) | 0,33 | 0 | 0 | 0 | 0 | 0 | 0,09 |
| **RUNX3** | 3 (43) | 1 | 1 | 0,15 | 0 | 1 | 0,07 | 0,04 |
| **RARB** | 0 (0) | 0,19 | 0 | 0 | 0 | 0 | 0 | 0 |
| **HLTF** | 0 (0) | 0 | 0 | 0 | 0 | 0 | 0 | 0 |
| **HLTF-2** | 0 (0) | 0,17 | 0,08 | 0,14 | 0,02 | 0 | 0,02 | 0,04 |
| **SCGB3A1** | 6 (86) | 0,27 | 1 | 1 | 0,63 | 0,66 | 1 | 0,79 |
| **SCGB3A1-2** | 6 (86) | 0,17 | 0,6 | 1 | 0,84 | 0,64 | 1 | 0,99 |
| **ID4** | 4 (57) | 0,71 | 1 | 1 | 0,04 | 0,83 | 0,06 | 0,01 |
| **ID4-2** | 0 (0) | 0 | 0 | 0 | 0,03 | 0,04 | 0,07 | 0,03 |
| **TWIST1** | 1 (14) | 0,02 | 0 | 0,09 | 0,03 | 1 | 0,07 | 0,02 |
| **SFRP4** | 2 (29) | 0,3 | 1 | 0 | 0,01 | 0,25 | 0,01 | 0 |
| **SFRP4- 2** | 0 (0) | 0,02 | 0,24 | 0,29 | 0 | 0 | 0,02 | 0 |
| **DLC1** | 1 (14) | 0,07 | 0,74 | 0,13 | 0,12 | 0,11 | 0,08 | 0 |
| **DLC1-2** | 1 (14) | 0 | 1 | 0,08 | 0,04 | 0 | 0,08 | 0,11 |
| **SFRP5** | 4 (57) | 0,11 | 1 | 1 | 0,02 | 0,3 | 0,95 | 0,26 |
| **SFRP5- 2** | 1 (14) | 0 | 1 | 0,03 | 0 | 0,03 | 0,27 | 0 |
| **BNIP3** | 2 (29) | 0,35 | 0,19 | 1 | 0,04 | 0,26 | 0,06 | 0,2 |
| **H2AFX** | 0 (0) | 0,01 | 0,1 | 0 | 0 | 0,01 | 0,03 | 0,06 |
| **H2AFX-2** | 0 (0) | 0,03 | 0,13 | 0,15 | 0,04 | 0,02 | 0,06 | 0,01 |
| **CCND2** | 4 (57) | 0,45 | 0,56 | 1 | 0,03 | 0,64 | 0,12 | 0,18 |
| **CCND2-2** | 5 (71) | 0,6 | 1 | 1 | 0,19 | 0,34 | 0,16 | 0,51 |
| **CACNA1G** | 1 (14) | 0,06 | 0,6 | 0,11 | 0,02 | 0,06 | 0,05 | 0,03 |
| **TGIF** | 0 (0) | 0 | 0 | 0 | 0 | 0 | 0 | 0 |
| **BCL2** | 0 (0) | 0,02 | 0 | 0 | 0 | 0,01 | 0,04 | 0 |
| **CACNA1A** | 5 (71) | 0,4 | 1 | 0,96 | 0,2 | 0,21 | 0,38 | 0,34 |
| **TIMP3** | 1 (14) | 0,02 | 0,48 | 0,25 | 0 | 0,17 | 0,03 | 0 |
| **TIMP3-2** | 2 (29) | 0 | 0,51 | 0 | 0,03 | 0,39 | 0 | 0 |
