# Supplementary material for: Multiplexed methylation profiles of tumor suppressor genes and clinical outcome in lung cancer
Source: J Transl Med. 2010 Sep 17;8:86. doi: 10.1186/1479-5876-8-86 (PMC2955578; doi:10.1186/1479-5876-8-86)
Supplement: Additional file 3 — Table S3: Complementary information of the genes analyzed using MS-MLPA. Review of the functional implications and methylation studies of the candidate genes analyzed in this study in lung cancer. [file 1479-5876-8-86-S3.DOC]

| **Gene**  **(alias)** | **Name^a^** | **Function** | **Lung cancer Methylation**  **References** |
| --- | --- | --- | --- |
| *PRDM2*  *(*RIZ, RIZ1, RIZ2, KMT8, MTB-ZF, HUMHOXY1) | PR domain containing 2, with ZNF domain | TSG, member of a nuclear hystone/protein methyltransferase superfamily, and methylates lys 9 of histone3. Involved in cell cylce control and apoptosis | Du, 2001 |
| *RUNX3*  (AML2, PEBP2A3) | Runt-related transcription factor 3 | Tumor suppresor function. Facilitates TGFb signaling and RUNX family was implicated in cell cycle regulation, differentiation, apoptosis and malignant transformation. | Yanagawa, 2003; Li, 2004; Sato, 2006; Licchessi, 2008; Jin, 2009; Yoshino 2009 |
| *RARB*  (HAP, NR1B2, RRB2) | Retinoic acid receptor beta | Mediates cellular signaling in embryonic morphogenesis, cell growth and differentation. Tumor supressor function due to its antiproliferative and pro-apoptotic effect | Chan, 2005; Feng, 2008; Hawes, 2009; Jin, 2009 |
| ***HLTF***  (HIP116A, HLTF1, RNF80) | Helicase-like transcription factor | Tumor supressor. Regulate transcription by altering the chromatin structure. Helycase and E3 ubiquitin-ligase activity. Involved in differentiation. | No references |
| *SCGB3A1*  (UGRP2, HIN-1, HIN1, LU105, PnSP-2) | Secretoglobin,  family 3A, member 1 | Tumor supressor. Growth inhibitory functions. Involved in epithelial differentation | Krop, 2004 and Shigematsu 2005 |
| ***ID4***  (bHLHb27) | Inhibitor of DNA binding 4, dominant negative helix-loop-helix protein | Involved in neurogenesis during embrionic life. Tumor supressor function but reports of tumor promotion | No references |
| *TWIST1*  (SCS, H-twist, BPES2, bHLHa38) | Twist homolog 1 (Drosophila) | Transcription factor. Involved in cell differentiation. Antiapoptotic and pro-metastatic roles | Yuan, 2004 |
| *SFRP4*  (frpHE, FRP-4, FRPHE) | Secreted frizzled-related protein 4 | Wnt pathway modulator. Pro-apoptotic properties. Cell growth and differentiation regulator | Tsou, 2007 and Licchessi, 2008 |
| *DLC1*  (HP, ARHGAP7, STARD12, DLC-1, p122-RhoGAP) | Deleted in liver cancer 1 | Tumor supressor activity.Involved in actin cytoskeleton structure regulation and focal adhesions and inhibition of cell growth. | Yuan 2004 and Dammann, 2005 |
| *SFRP5*  (SARP3) | Secreted frizzled-related protein 5 | Wnt pathway modulator Cell growth and differentiation regulator. Involved in determining the polarity of photoreceptor cells in the retina. | Licchesi, 2008; Yoshino, 2009; Tsou, 2007 and Suzuki, 2010 |
| ***BNIP3***  (Nip3) | BCL2/adenovirus E1B 19kDa Interacting protein 3 | Member of BCL2 family. Involved in apoptosis and autophagia. Induced by HIF (hipoxia inducible factor). | No references |
| ***H2AFX*** | H2A hsitone family, member X | Responsible of nucleosome structure of the chromosomal fiber in  eukaryotes | No references |
| *CCND2* | Cycline D2 | Implicated in cell cycle regulation (phase G1/S transition), differentiation and malignant transformation. Inhibit cell proliferation | Virmani, 2003; Feng, 2008; Hawes, 2009 and Kubo, 2009 |
| ***CACNA1G***  (Cav3.1, NBR13) | Calcium channel, voltage-dependent, T type, alpha 1G subunit | Mediate the entry of calcium ions into excitable cells. Involved in including muscle contraction, hormone or neurotransmitter  release, gene expression, cell motility, cell division and cell death. | No references |
| ***TGIF*** | #TGFB-induced factor homebox 1 | Transcriptional corepressor of SMAD2. TGIF protein has a role in inhibiting 9-cis-retinoic acid-dependent RXR alpha transcription activation of  the retinoic acid responsive element | No references |
| ***BCL2***  ***(Bcl-2***) | B-cell CLL/lymphoma2 | Anti-apoptotic properties and essential role in autophagy | Nagakate, 1996 |
| ***CACNA1A***  (Cav2.1, EA2, APCA, [HPCA](http://www.genenames.org/cgi-bin/hgnc_search.pl?field=symb_prev_and_alias&andnot=AND&search_type=simple&anchor=equals&sortby=symbol&match=HPCA), FHM) | Calcium channel, voltage-dependent, P/Q type, alpha 1A subunit3 | Mediate the entry of calcium ions into excitable cells. Involved in including muscle contraction, hormone or neurotransmitter  release, gene expression, cell motility, cell division and cell death | No references |
| *TIMP3* | TIMP metallopeptidase inhibitor 3 | Tumor supressor function. Inhibit matrix metalloproteinases. Pro-apoptotic and anti-angiogenic properties | Dammann, 2005; Gu, 2006 and Wang, 2008 |

a. Human Genome Organization Nomenclature

b. Approved gene name from Human Genome Organization website http: //www.genenames.org/
